# Long-term effects of cognitive behavioural therapy on the quality of life in adult ADHD

**DOI:** 10.1371/journal.pone.0356825

**Published:** 2026-08-28

**Authors:** Ravian Wettstein, Valentina Navarro-Ovando, Viktoria Janke, Ron Mathôt, Glenn Dumont

**Affiliations:** 1 Dedimo, Research and Development, Utrecht, The Netherlands; 2 Department of Hospital Pharmacy and Clinical Pharmacology, Amsterdam University Medical Centre, Location AMC, Amsterdam, The Netherlands; Taipei Veterans General Hospital, TAIWAN

## Abstract

This study examined the long-term effects (4–8 years) of Cognitive Behavioural Therapy (CBT), added to pharmacotherapy and psychoeducation (PE), on quality of life (QoL) in adults with Attention-Deficit/Hyperactivity Disorder (ADHD). Although previous findings showed no added QoL benefit of CBT immediately post-treatment, CBT provides skill-building and coping strategies that may yield delayed benefits in terms of ADHD-related functional impairments. We hypothesize that CBT will provide delayed treatment gains and thus results in higher QoL at follow-up or a slower decline in QoL over time. A follow-up study was conducted with 627 adults from a prior cohort that evaluated short-term CBT outcomes. Of these, 159 participants completed a long-term follow-up survey assessing QoL using the Adult ADHD Quality of Life scale (AAQoL). A linear mixed model revealed no significant treatment or treatment-by-time effects for the total AAQoL or its subdomains. However, a significant effect of time was observed across all measures. Post hoc comparisons indicated an increase in QoL from baseline (pooled mean = 44.4; PsyEd: 46.4, PsyEd + CBT: 42.4) to post-treatment (69.3), followed by a decline at follow-up (52.1), which remained above baseline levels. No significant between-group differences were found in the proportion of participants achieving clinically meaningful QoL improvements over time. These findings suggest that this study does not provide evidence for an added long-term QoL benefit of CBT beyond PE and pharmacotherapy, whereas initial treatment was associated with sustained improvement. Continued care beyond initial treatment may help maintain gains in adults with ADHD.

## Introduction

Attention Deficit Hyperactivity Disorder (ADHD) is characterized by persistent patterns of inattention, hyperactivity, and impulsivity that may span the entire lifespan. In adults, the global prevalence is estimated to range between 2.6% and 3.6% [1]. Functional impairments associated with ADHD negatively affect various life domains, including social participation, academic and occupational development, and (interpersonal) relationships, all of which contribute to a diminished quality of life (QoL) [2–6]. ADHD related functional impairments also increase the risk of developing negative beliefs regarding self-esteem and self-efficacy, contributing to the development of dysfunctional behaviours and increasing the risk of comorbidities like anxiety and depression, further reducing the QoL of adults with ADHD [7,8].

Quality of life is defined by the World Health Organization as an individual’s perception of their position in life in the context of their culture and value systems, and in relation to their goals, expectations, standards and concerns [9]. Adults with ADHD report lower QoL scores compared to their non-ADHD peers across various life stages and even after undergoing treatment [5,6,10–12], with all QoL domains affected [13]. QoL in adults with ADHD declines with age, and in the presence of comorbidities [10,14,15]. The severity of ADHD symptoms and associated deficits in executive functions — particularly poor working memory and emotional dysregulation — appear to have the greatest impact on QoL [4,5,11,13].

In clinical practice, managing adult ADHD effectively is recommended to rely on a multimodal approach combining pharmacological and non-pharmacological interventions [16–18]. Although pharmacotherapy is effective in reducing core symptom severity [17,19], its isolated impact on long-term QoL remains limited. A recent meta-analysis by Bellato et al. (2025) found that pharmacological effects on QoL typically demonstrate only small effect sizes [20], and medication discontinuation does not appear to significantly impact QoL in adults, unlike in younger populations [21]. These findings suggest that while pharmacotherapy is essential for acute symptom control, integrating non-pharmacological strategies — such as psychoeducation and Cognitive Behavioural Therapy (CBT) — is recommended for achieving clinically meaningful, sustained improvements in daily functioning and overall QoL [2,22].

Psychoeducation for ADHD generally aims to provide patients and their relatives with information about the characteristics of ADHD to enhance insight into its symptoms, causes, treatment options, and coping strategies [23]. However, beyond this educational focus, there is no consensus on its format, content, or the qualifications required of those who deliver it [24,25]. CBT, by contrast, has a primary focus on restructuring dysfunctional cognitive mechanisms and behaviours while providing compensating strategies to overcome ADHD symptoms [26]. Although most CBT protocols include a psychoeducational module, their primary goal is to equip patients with skills to manage daily functional impairments. The heterogeneity of psychoeducation as a construct and its potential overlap with the educational components of CBT complicate the interpretation of between-group comparisons, as differences in outcomes may partly reflect differences in intervention content rather than the specific active ingredients of CBT [23]. Furthermore, since psychoeducation focuses on improving understanding and insight about ADHD without systematically targeting the development of coping strategies, its effects may be less durable than those of CBT — though this assumption requires empirical verification, particularly over longer follow-up periods.

CBT appears as the most studied non-pharmacological approach and shows the strongest empirical support compared to other non-pharmacological interventions [27]. CBT reduces the severity of ADHD symptoms, improves daily functioning and reduces the occurrence or severity of comorbidities like anxiety, depression, and internalized symptoms [16,27–29]. Among the mechanisms through which CBT exerts its effects, improvements in self-efficacy and self-esteem are considered particularly relevant, as dysfunctional beliefs related to these constructs are central targets of CBT adapted for adults with ADHD [7,8,30]. The combination of CBT and medication has been shown to be more effective than either medication alone or CBT alone in reducing ADHD symptoms and improving functionality in adults diagnosed with ADHD [22,31,32].

Despite the strong evidence for CBT on symptom reduction, its effect on QoL remains poorly established. A Cochrane review by López et al. (2018) found that only 5 of 14 included RCTs reported QoL as an outcome [22], highlighting the limited evidence base. Among studies that do include QoL, findings are inconsistent, likely reflecting important methodological differences. Studies vary in the type of control condition — ranging from waitlist controls to active psychoeducation or treatment as usual — which directly influences the likelihood of detecting between-group differences [22,33–35]. QoL has been operationalised using different instruments across studies, including disorder-specific measures such as the AAQoL and abbreviated generic measures such as the psychological domain of the WHOQOL-BREF [36,37], limiting cross-study comparability. Follow-up durations also vary substantially, from weeks to over a year, making it difficult to draw conclusions about the durability of treatment effects [34,35,37].

To date, no study has examined whether CBT yields delayed benefits on QoL beyond one year post-treatment in adults with ADHD, despite evidence that ADHD is associated with persistent functional impairments across the lifespan [38]. This represents a critical gap in the literature, as the skill-building nature of CBT suggests that its effects may accumulate over time — consistent with evidence that CBT benefits on symptoms and functioning can persist for up to one to two years post-treatment [37,39]. Longitudinal follow-up of the same participants provides the strongest possible basis for examining delayed treatment effects, as it avoids selection bias associated with new recruitment and enables direct within-person comparison over time. To this end, we conducted a follow-up study of a previously described cohort of adults with ADHD [40]. Hence, the aim of this study was to investigate the long-term effects (4–8 years after treatment) of pharmacotherapy and psychoeducation, with or without CBT, on the QoL in adults with ADHD. The follow-up period of 4–8 years represents one of the longest follow-up intervals reported in this literature, and QoL was assessed using the AAQoL, enabling direct comparison with the previously reported absence of short-term QoL benefit. We hypothesize that the acquisition of skills and tools for managing ADHD-related functional impairments through CBT will provide treatment gains over a longer time period compared to psychoeducation alone, resulting in higher QoL at follow-up or a slower decline in QoL over time. Answering this question has direct clinical implications, as it informs decisions about whether CBT should be recommended as part of long-term ADHD management alongside pharmacotherapy and psychoeducation.

## Materials and methods

### Design

This study is a follow-up from a previous observational cohort study focused on assessing the acute effects of CBT on QoL in ADHD patients as an addition to treatment as usual (medication in combination with psychoeducation) [40].

All participants from the cohort study were invited between 30^th^ of August and 23th of September 2022 for a follow-up assessment. In total, 627 participants were invited, from which 305 belonged to the medication + psychoeducation only (PsyEd) group and 322 to CBT along medication + psychoeducation (PsyEd + CBT) group.

The study received approval of the medical-ethical commission of the Amsterdam Medical Center on the 13^th^ of July 2022 and was registered in clinicaltrials.gov with the identifier NL79674.018.22.

### Participants

All participants were adults that underwent their diagnostic and intervention process for ADHD at ADHDcentraal and participated in the initial study [37]. ADHDcentraal is a multicentre mental health care facility specialized in the diagnostics and treatment of adult ADHD, with participants recruited from five locations across the Netherlands All participants were assessed by a team consisting of a psychologist, psychiatrist and a nurse practitioner specialized in ADHD. After establishing the diagnosis, participants were provided with information about CBT, which was offered as an optional treatment.

### Treatment protocol

#### Pharmacological treatment.

All participants were treated according to the Dutch guideline for ADHD in adults [41]. Follow up sessions with a clinician were scheduled every two to three weeks to assess further treatment adaptations until optimal pharmacotherapeutic improvement was obtained.

#### Psychoeducation.

All participants with a confirmed diagnosis of ADHD received at least one session of psychoeducation. During psychoeducation information was provided about the condition, related functional challenges and the specific implications of the pharmacological treatment of ADHD and were guided by a trained nurse practitioner.

#### CBT.

The first CBT session was provided within one month after the establishment of the ADHD diagnosis. CBT sessions lasted 45–60 minutes and were provided based on the guidelines published by Safren et al. (2004, 2005) [30,42]. The sessions were oriented to provide tools and strategies to overcome functional limitations and dysfunctional beliefs associated with ADHD. The themes included time management, planning, organization, emotional recognition and regulation. The aim of CBT was tailored to the individual’s needs. All sessions were guided by a certified clinical psychologist with training in CBT. The number of sessions varied from 5 to 16, with the patient and treating psychologist jointly determining when treatment goals were sufficiently achieved. Variability in session number may reflect differences in the rate at which individual treatment goals were met, early discontinuation, or practical constraints such as time availability or travel distance. All participants received the core modules of the CBT protocol, comprising organisation and planning, coping with distractibility, and cognitive restructuring. Optional modules — including procrastination, anger management, and communication skills — were addressed based on individual needs and available treatment time.

### Procedure

Participants were invited to participate in this study via email. Participants who agreed to participate were contacted via phone to explain the study and provided written consent. A gift card was offered as a compensation for their participation.

Data was collected using the online-survey module of Castor EDC (EDC, Castor). The survey was divided into five sections: Demographic and intervention information, the Adult ADHD Quality of Life (AAQoL), the Adult ADHD Self-Report Scale (ASRS v1.1), the Rosenberg Self-Esteem Scale (RSES) and the General Self-Efficacy Scale (GSES). The estimated time for completion of the survey was 30 minutes.

### Questionnaires/Outcome measures

#### Quality of life (QoL) - Adult ADHD Quality of Life (AAQoL).

The AAQoL scale is a scale that assesses quality of life in adults with ADHD. The AAQoL scale consists of 29 items that are rated on a 5-point Likert scale. The AAQoL scale has four subscales: Life productivity (LP), life outlook (LO), relationships (R), and psychological health (PH). The AAQoL is validated in the US and European population with similar psychometric properties [3,43]. The AAQoL scale has shown adequate internal consistency/reliability (Cronbach’s alpha = 0.74 to 0.93 [3]. Convergent validity results present moderate correlations with CAARS and CGI-S scores, and strong correlation with BRIEF-A scores [43]. The discriminant validity has been confirmed in comparison to CGI-S scores. AAQoL scores were negatively associated with CGI-S scores, indicating a reduced quality of life with increased severity of the disease [43].

#### ADHD symptoms’ severity – Adult ADHD Self-Report Scale, first edition (ASRS v.1.1).

The ASRS v1.1 is a self-report scale for the severity of ADHD symptoms in adults. The scale consists of 18 items that are rated on a five-point Likert scale. Based on the total score, the severity of ADHD symptoms can be interpreted as Mild (between 0–18 points), Moderate (between 19–20 points) or Severe (more than 21 points). The ASRS v1.1 scale is developed in relation to the DSM-IV criteria of ADHD. The scale has an internal consistency of 0.76 to 0.88, a sensitivity of 71% and a specificity of 67–77% [44]. The scale takes approximately five minutes to complete.

The scale was translated from English into Dutch by two investigators. To find possible translation discrepancies, two other investigators translated the Dutch version back into English and discussed any discrepancies for purposes of clarity.

#### Self-Efficacy - General Self-Efficacy Scale (GSES).

The GSES measures the concept of generalized self-efficacy, characterized by a “broad and stable sense of personal competence and coping effectively with diverse stressful situations”. The scale consists of 10 items that are rated on a four-point Likert scale. No cutting points are considered by the authors [45]. A higher score represents a higher representation of self-efficacy. The GSES Dutch translation was used [46]. The GSES has a high internal consistency (Cronbach’s alpha = 0.76 to 0.90) and test-retest correlation (r = 0.74 to 0.78) [47].

#### Self-Esteem – The Rosenberg Self-Esteem Scale (RSES).

The RSES scale assesses global self-esteem, measuring “a favourable or unfavourable attitude towards the self” [47]. The scale has been translated into Dutch by Franck et al. (2008) [48]. A total score of 18 or higher equals a good self-esteem, 15–18 points equals a low self-esteem, and a score of lower than 15 points is equal to a very low self-esteem. The Dutch translation of the GSES has a high internal consistency (Cronbach’s alpha = 0.86 to 0.89) [49]. Construct validity of the RSES has been proven in comparison to the Big Five personality traits: RSES scores are positively correlated with extraversion and conscientiousness and negatively correlated with neuroticism [49]. Factor analysis suggested a one-factor structure of the scale, in line with the unidimensional scale [49].

### Statistical analysis

Data was analysed using R version 4.2.3. Parts of the data collected in the original study [40] were included for the main analysis. Specifically, the measures of AAQoL obtained before (Baseline) and after treatment (Post), hours of treatment, and the dates of intake and end of treatment. The last was used to calculate the time after discharge until follow-up (FU). The hours of treatment refer to the consultation time registered by the treating clinicians, and represent all modules of treatment (PsyEd, CBT, and pharmacotherapy).

To assess potential attrition bias, respondents were compared with non-respondents on available baseline characteristics. Independent-samples t-tests were used for continuous variables (total, LP, PH, LO, and R AAQoL score and age) and chi-squared tests for categorical variables (sex and treatment group). Additionally, a logistic regression model was fitted with response status as the outcome and all baseline characteristics as predictors.

The main objective, the added value of CBT on the long-term QoL (AAQoL), was analyzed using a linear mixed-effect model (LME; with nlme package version 3.1–163), with treatment, time, and treatment-by-time as fixed effects, and participants and time as random effects. Time was included as random effect to account for potential correlations between measurements taken at different time points within the same individual. Fixed effect estimates are reported along the degrees of freedom F-statistic, p-value, and marginal *R*^*2*^ (*R*^*2*^m, i.e., proportion of variance explained by the fixed effects) and conditional *R*^*2*^ (*R*^*2*^c, i.e., proportion of variance explained by both the fixed and random effects) [50]. Model assumptions were examined through inspection of residual plots and Q-Q plots of the random effects. The same procedure was repeated for each of the AAQoL subscales, except for Life Productivity, where this model did not converge; a random-intercept-only model was therefore used for this subdomain. In case of significant fixed effects, pairwise comparisons were conducted with Bonferroni corrections of multiple testing.

Cohen’s d with 95% confidence intervals was calculated for between-group differences at each time point using the *effectsize* package (version 1.0.1) in R.

Between group differences for the ADHD-RS, GSES, and RSES scores were assessed using two-sided independent t-tests.

Clinical relevance of the AAQoL scores was evaluated, by calculating the contrasts between the three different time points, and a threshold of 8 points in the AAQoL was used as suggested by Tanaka et al. (2019) for clinical relevance [51]. Differences were calculated as follows: FU-Post; FU-Baseline; Post-Baseline. Differences in the total AAQoL score of ±8 will be considered as ‘Decline’ or ‘Improvement’ based on its direction, while differences smaller than 8 are considered as ‘No-change’. Multiple ordinal regression method was used (MASS package version 7.3–60) to assess whether treatment, age, sex, total hours of treatment, and adherence to pharmacotherapy were associated with a relevant clinical improvement of QoL.

Pearson correlation analysis (stats package version 4_4.2.3) was used to assess the effect of FU time as a continuous variable (months after discharge) on the change in AAQoL between discharge and FU.

Alpha was set at 0.05 for all analysis, except when using Bonferroni corrections of multiple testing.

To evaluate the sensitivity of the study to detect clinically meaningful between-group differences, a retrospective power analysis was conducted using the R pwr package (version 1.3.0).

## Results

### Sample characteristics

A total of 159 participants completed the questionnaires, representing approximately 25% of those invited (n = 627). Attrition analyses revealed no significant differences between respondents and non-respondents on any available baseline characteristic. Full results of the attrition analysis are presented in Supplementary Table S1 in S1 File. Sample characteristics and pairwise comparisons between groups are presented in Table 1. The groups did not differ significantly in terms of size, sex distribution, age, average time since discharge, or pharmacotherapeutic adherence. In both groups; PsyEd 57% and PsyEd + CBT, 50% discontinued medication at some point between discharge and follow-up. The PsyEd group received a mean of 15 hours of treatment (SD = 2), whereas the PsyEd + CBT group received 32 hours (SD = 3.2), including on average 12 hours of CBT (SD = 2). Groups differed significantly in total treatment hours (P < 0.001), but this difference was not significant after adjustment for the CBT hours (P = 0.064).

**Table 1:** Sample characteristics.

	PsyEd	PsyEd + CBT	*P*
N	81	78	
Sex			0.681
N (%) Females	41 (51%)	43 (55%)	
Mean Age (SD)	34 (12)	31 (10)	0.061
Mean hours of treatment (SD)	15(2)	32(3)	<0.001*
Mean number of CBT sessions (SD)	–	12(2)	–
Mean number of months after discharge (SD)	75(10)	74(10)	0.579
Adherence to pharmacotherapy (%)	44 (57%)	39 (50%)	0.699

*Group differences were no longer significant after adjusting for CBT hours.

### Total AAQoL

**Fig 1:**
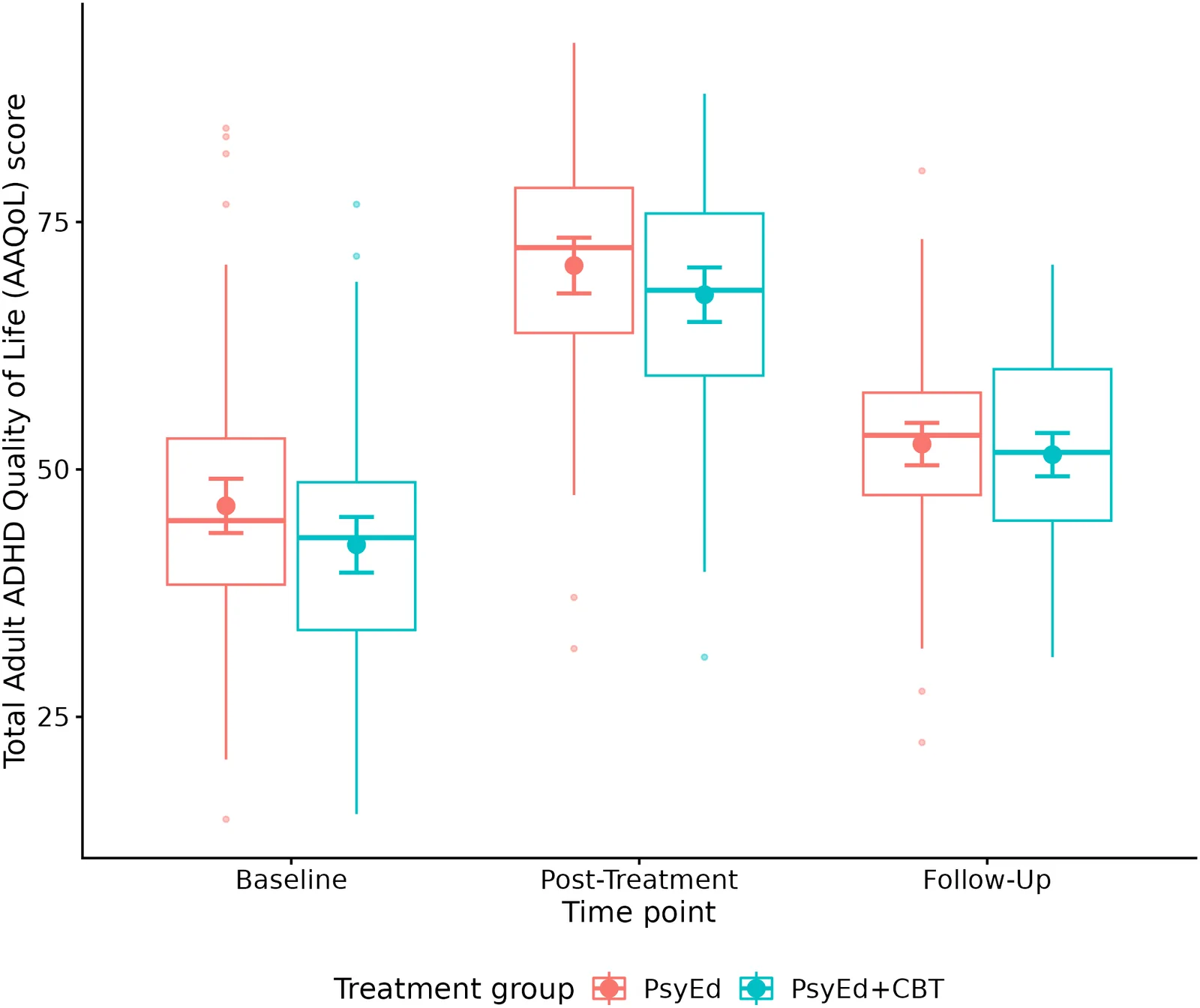
Total Adult ADHD Quality of Life scores between groups over time. *Note.* Boxplots display the Total Adult ADHD Quality of Life (AAQoL) scores for the PsyEd (n = 81) and PsyEd + CBT (n = 78) groups at baseline, post-treatment, and follow-up. The horizontal line within each box represents the median, and the whiskers indicate the interquartile range (IQR). Individual points represent outliers. Filled circles and error bars represent model-estimated means and 95% confidence intervals derived from the linear mixed-effects model. Higher scores indicate a better quality of life. PsyEd + CBT = cognitive behavioural therapy, psychoeducation, and pharmacotherapy; PsyEd = psychoeducation and pharmacotherapy alone.

For the main analyses, data from 14 participants that could not be collected in the original study were included. Inspection of missing data patterns revealed that missingness was concentrated among females in the PsyEd group at post-treatment (14.6%), suggesting that Missing Not at Random cannot be fully excluded. Mean total AAQoL scores for each group at different time points are shown in Fig 1.

The estimated fixed effects for all AAQoL measures are presented in Table 2. At baseline, the mean total AAQoL was 46.38 (SD = 12.8) for PsyEd and 42.41 (SD = 11.9) for PsyEd + CBT. Post-treatment the mean total AAQoL was 70.8 (SD = 12.8) for PsyEd and 67.7 (SD = 11.7) for PsyEd + CBT, while at FU a mean total AAQoL of 52.6 (SD = 9.9) and 51.5 (SD = 9.6) was found, respectively.

**Table 2:** Estimated Fixed Effects for total Adult ADHD Quality of Life and its subdomain scores.

AAQoL scores	Effect	numDF	denDF	F	*P*	*R* ^*2*^ *m*	*R* ^*2*^ *c*
Total						0.45	0.89
	Treatment	1	157	2.48	0.117		
	Time	2	300	191.23	<0.001		
	Treatment x Time	2	300	0.94	0.389		
Life productivity						0.37	0.48
	Treatment	1	157	2.66	0.105		
	Time	2	300	72.17	< 0.001		
	Treatment x Time	2	300	0.47	0.623		
Psychological health						0.25	0.86
	Treatment	1	157	3.40	0.067		
	Time	2	300	52.99	< 0.001		
	Treatment x Time	2	300	0.17	0.845		
Life outlook						0.36	0.88
	Treatment	1	157	1.21	0.22		
	Time	2	300	94.33	< 0.001		
	Treatment x Time	2	300	1.15	0.317		
Relations						0.37	0.84
	Treatment	1	157	1.62	0.206		
	Time	2	300	32.82	< 0.001		
	Treatment x Time	2	300	1.71	0.183		

*Note.* AAQoL = Adult ADHD Quality of Life; numDF = numerator degrees of freedom; denDF = denominator degrees of freedom; *R*^*2*^m = marginal *R*^*2*^ (variance explained by fixed effects); *R*^*2*^c = conditional *R*^*2*^ (variance explained by both the fixed and random effects). R²c values are not directly comparable across subdomains: the Life Productivity model included a random intercept only (the random slope for time did not converge), whereas the Psychological Health, Life Outlook, and Relations models included both a random intercept and a random slope for time, which inflates R²c independently of model fit. Statistical significance is indicated by P < .05.

The LME analysis revealed no significant treatment-by-time effect on total AAQoL score, nor a significant effect of treatment. Between-group effect sizes were small at baseline (d = 0.32, 95% CI [0.00, 0.64]), small at post-treatment (d = 0.25, 95% CI [−0.07, 0.57]), and negligible at follow-up (d = 0.11, 95% CI [−0.20, 0.42]). Inspection of model assumptions indicated approximate normality of the residuals with minor heteroscedasticity, and satisfactory normality of the random intercept. More pronounced deviations were observed for the random slopes, attributable to a small subset of participants showing little or no treatment response. Full assumption plots are presented in Supplementary Figure S1 in S1 File. The retrospective power analysis indicated that the study had 99.9% power to detect a between-group difference equal to the minimal clinically relevant difference of 8 AAQoL points (d = 0.82). The effect of time on the Total AAQoL score was significant (P < 0.001). Post hoc pairwise comparisons revealed a significant increase in total AAQoL scores post-treatment (t(302)=−17.4, P < 0.001) followed by a significant decline at FU (t(284)=13.5, P < 0.001). Despite this decline, total AAQoL scores at follow-up remained significantly higher than those measured before initiation of treatment (*t*(290)=*−5.9*, P < 0.001).

A sensitivity analysis using multiple imputation yielded results consistent with the primary analysis, with no significant treatment or interaction effects (Supplementary Table S4 in S1 File).

### AAQoL subdomains

For all subdomains — Life Productivity, Psychological Health, Life Outlook, and Relationships — only a significant main effect of time was observed (all P < 0.001), with no treatment or interaction effects. Post hoc pairwise comparisons across time points followed a similar temporal pattern for all subdomains, consistent with that observed for the total AAQoL score. A full summary of post hoc results is presented in Supplementary Table S2 in S1 File.

### Comparison of self-efficacy, self-esteem, and symptom severity

In the PsyEd group, self-efficacy was 31.9 (SD = 4.8) and comparable to the PsyEd + CBT group 31.2 (SD = 4.5). Self-esteem was also comparable, revealing an average of 26.8 (SD = 2.4) for the PsyEd group and 27.2 (SD = 1.9) for the PsyEd + CBT group. The ADHD symptom severity was 9.3 (SD = 3.9) in the PsyEd group, and 9.7 (SD = 3.3) in the PsyEd + CBT group and non-significant.

### Clinically relevant improvement of QoL

In terms of clinically relevant improvement, in the PsyEd group at FU 57 declined, 14 showed no change, and two improved when compared with discharge, while in the PsyEd + CBT 57 showed a decline, 14 showed no change and six improved at FU. At FU in comparison to baseline, in the PsyEd group nine showed a decline, 34 showed no change and 36 improved, while in the PsyEd + CBT five declined, 30 showed no change and 40 improved at FU. The multiple ordinal regression revealed no significant association of treatment, sex, age, total hours of treatment, or adherence to pharmacotherapy on clinically relevant improvement scores (Supplementary Table S3 in S1 File).

The FU time was significant and negatively correlated with Total AAQoL (*r = −.22, t*(148)=−2.74, P = 0.007) regardless of group, although negligible. For the subscales, only a significant correlation (negligible) between FU time and life productivity was found (r = −.22, t(148)=−2.71, P = 0.008).

## Discussion

This follow-up study aimed to assess the long-term added value of CBT to care as usual on the quality of life of adults diagnosed with ADHD, 4–8 years after discharge. To our knowledge, this study is the first examining the long-term effects of CBT on quality of life in this population beyond one-year post-treatment.

Contrary to our hypothesis, no significant long-term differences in AAQoL scores were observed between psychoeducation alone versus psychoeducation combined with CBT. Within the constraints of the current observational design, these findings do not provide evidence for an added short- or long-term QoL benefit of CBT beyond care as usual.

None of the AAQoL subdomains — Life Productivity, Psychological Health, Life Outlook, or Relationships — showed a significant main effect of treatment or a significant treatment-by-time interaction; only a significant effect of time was observed across all subdomains. These findings are consistent with previous studies reporting no effect of CBT on QoL when delivered alongside psychoeducation (and medication) in adults with ADHD [52]. However, direct comparisons across studies are complicated by important methodological differences. Studies vary considerably in the type of control condition used — ranging from waitlist controls to active psychoeducation or treatment as usual — which directly influences the likelihood of detecting between-group differences. Furthermore, QoL has been operationalised using different instruments across studies, including the AAQoL [40], abbreviated versions of the WHOQOL-BREF [36], and disorder-specific measures, limiting cross-study comparability. Follow-up durations also vary substantially, from weeks to years, making it difficult to draw conclusions about the durability of treatment effects. These methodological differences may partly explain the inconsistent findings across studies [22,52]. Moreover, the absence of evidence for an added benefit of CBT beyond psychoeducation does not appear unique to ADHD. Similar patterns have been reported in other mental health conditions, including depression [53,54], bipolar disorder [55], and schizophrenia [56,57].

The lack of a clear effect raises important questions about the active components of CBT and psychoeducation and how they contribute to treatment outcome. For example, a recent review by Pedersen et al. (2024) highlights the heterogeneity of psychoeducational interventions for ADHD, emphasizing the absence of a unified definition or format [23]. This ambiguity has led to inconsistent classification in research, where psychoeducation is sometimes used as an active control [58] and at other times considered a stand-alone behavioural intervention, which may obscure possible CBT benefits [27]. Although CBT protocols for ADHD are generally more clearly defined, they still exhibit substantial variation in content and delivery, falling short of a unified, standardized approach [27]. Such heterogeneity may contribute to the absence of observed differences when CBT is compared with other psychological interventions. Moreover, limited effects of CBT have been documented even when interventions are rigorously classified. For example, Baardseth et al (2013) consulted 91 CBT experts to categorize adult anxiety interventions as CBT or non-CBT; the authors found no significant differences between ‘confirmed’ CBT and non-CBT approaches on either disorder-specific or general symptoms [59].

Thus, we argue that the methodology of both CBT and psychoeducation should be defined and described more clearly, in order to be able to assess which elements are clinically meaningful. However, given that psychoeducation combined with pharmacotherapy is less time-consuming and does not require specialist psychological expertise, and given that the current study does not provide evidence for an added benefit of CBT on QoL, psychoeducation may represent a pragmatic option for enhancing QoL in adults with ADHD — at least until more definitive evidence from randomised controlled trials becomes available.

Finally, current results show that QoL scores remained significantly elevated post-treatment compared to pre-treatment. However, a general decline in QoL was observed over the follow-up period, indicating that the initial treatment gains were not fully maintained. Although the precise reasons for this decline cannot be determined from the available data, the most parsimonious explanation is the absence of continued clinical support following discharge. This is consistent with the understanding that ADHD is a lifelong condition for which sustained management may be necessary [60,61], and suggests that initial treatment alone may be insufficient for long-term QoL maintenance in adults with ADHD.

The major limitation of this study is that the allocation of the patients was not randomized but dependent on the choice made by the patient after receiving the diagnosis. As a real-world observational study conducted within routine clinical practice, this design enhances the ecological validity of the findings — reflecting how treatment decisions are made in everyday care — but inherently limits the control of potential confounders related to treatment type. In the clinical context of ADHDcentraal, practical factors such as time constraints, travel distance, and occupational demands are likely to have influenced treatment choice for a substantial proportion of patients. Although findings from the original cohort study suggested potentially greater symptom burden in the PsyEd + CBT group at baseline [40], post-hoc explorative comparisons in the current follow-up sample did not reveal statistically significant between-group differences on any available baseline characteristic, including the Psychological Health subdomain (t(151.4) = 1.89, p = .061). Comorbidity data were unavailable, however, had the PsyEd + CBT group carried a substantially greater comorbidity burden — comorbid psychiatric conditions such as anxiety and depression being well-established contributors to reduced quality of life in adults with ADHD [12] — this would plausibly have manifested as lower baseline QoL scores, which were not observed in the current sample. This baseline comparability on measured characteristics provides some reassurance, but does not rule out residual confounding from other unmeasured comorbidities, such as autism spectrum disorder or substance use disorders. Because this cohort was not selected on the basis of comorbidity status, it is likely broadly representative of the heterogeneous clinical population of adults with ADHD; however, we cannot exclude the possibility of effect modification across comorbidity subgroups, meaning that the average null effect reported here may mask subgroup-specific treatment effects that future, adequately powered studies with baseline comorbidity characterization should investigate. A separate limitation concerns the attrition analysis. Although this demonstrated no significant differences between respondents and non-respondents on available baseline characteristics, baseline ADHD symptom severity could not be included in this comparison, as these data were not available for the full original cohort. This represents a remaining limitation of the attrition analysis. Secondly, the hours of treatment varied within each group. Although the number of CBT sessions was recorded, and total treatment hours were included as a covariate in the analysis of clinically relevant improvement, total treatment hours reflect combined consultation time across CBT, psychoeducation, and pharmacological management, and therefore do not isolate the specific contribution of CBT dose. A dose-response analysis restricted to the number of CBT sessions was not performed, as this measure is only defined within the PsyEd + CBT arm and its limited range would provide insufficient power to detect a dose-response relationship. As the combination of psychoeducation and pharmacological management was comparatively less time-consuming, we do not think this conflation meaningfully affects our overall conclusions, though the specific dose-response relationship of CBT itself remains unexamined. Furthermore, no standardised psychoeducation protocol was used, and detailed information regarding the duration and content structure is unavailable. This limits the characterisation of the active control condition and is consistent with the broader heterogeneity of psychoeducation interventions documented in the literature [23]. As psychoeducation for adult ADHD commonly covers themes such as coping and psychological skills, the extent of overlap with the behavioural components of CBT could not be determined in the current study. Finally, considering the extended time frame between discharge and the follow-up, it was not possible to rule out that patients received additional psychological treatments or made changes to their pharmacotherapy during this period, as no data on post-discharge treatment were collected. Such uncontrolled intervening treatments represent a potential confound that could have attenuated or obscured between-group differences. However, pharmacotherapy adherence at follow-up was comparable between groups, providing at least partial evidence that the groups did not diverge substantially in their treatment trajectories during the follow-up period.

In conclusion, results from this study do not provide evidence for an added value of CBT in terms of QoL over psychoeducation combined with pharmacotherapy for adults with ADHD assessed 4–8 years after discharge from treatment. Generally, QoL scores were the highest directly after treatment, and declined thereafter, although QoL scores remained higher than before treatment. These findings are broadly consistent with current clinical guidelines, which recommend pharmacotherapy as the secondary treatment for adults with ADHD with significant functional impairment, with psychoeducation as the initial psychosocial intervention [17,18,41]. CBT is recommended as an adjunctive intervention for patients with persistent functional impairment or when pharmacotherapy is not feasible or preferred. The current results support this stepped-care approach, suggesting that for many patients, psychoeducation combined with pharmacotherapy may be sufficient to achieve meaningful short-term QoL improvements, while the observed long-term decline — consistent with evidence that ADHD treatment effects may diminish over time [61] — highlights the potential need for continued clinical support beyond the initial treatment episode. Future research should examine which forms of follow-up care — such as booster sessions, periodic monitoring, or stepped-care approaches — are most effective in maintaining QoL gains in adults with ADHD.

## Supporting information

S1 File Supplementary online content (Tables S1-4 and Figure S1. Table S1:Attrition Analysis. Block 1 presents descriptive statistics and between-group comparisons on continuous variables (independent-samples t-tests) and categorical variables (chi-squared tests). Block 2 presents logistic regression results with response status as the outcome variable. Table S2: Post-hoc Pairwise Comparisons of AAQoL Subdomain Scores Across Time Points (LP, PH, LO, R). Table S3: Multiple Ordinal Regression: Predictors of Clinically Relevant Quality of Life Improvement. Table S4: Sensitivity Analysis: Linear Mixed-Effects Model Fixed Effects Estimates Following Multiple Imputation (m = 20). Figure S1: Diagnostic Plots for Linear Mixed-Effects Model Assumptions: Residuals and Random Effects. Panel A: Fitted vs Residuals plot. Panel B: Q-Q plot of model residuals. Panel C: Q-Q plot of random effects for the intercept. Panel D: Q-Q plot of random effects for Time After.
(DOCX)
