# Supplementary material for: Long-term effects of cognitive behavioural therapy on the quality of life in adult ADHD
Source: PLoS One. 2026 Aug 28;21(8):e0356825. doi: 10.1371/journal.pone.0356825 (PMC13524267; doi:10.1371/journal.pone.0356825)
Supplement: Supplementary file 1 [file pone.0356825.s001.docx]

Supplement information

**Table S1.** Attrition Analysis: Comparison of Respondents and Non-Respondents on Baseline Characteristics and Logistic Regression of Response Status Predictors

| **Block 1. Baseline Characteristics by Response Status** | | | | | |
| --- | --- | --- | --- | --- | --- |
| **Characteristic** | **Non-respondents(n = 461)** | **Respondents (n = 159)** | **Test statistic** | **df** | **p** |
| **Continuous variables (independent-samples t-test)** | | | | | |
| Baseline total AAQoL, M (SD) | 43.7 (13.0) | 44.5 (12.5) | t = 0.66 | 276.2 | .511 |
| Baseline LP AAQoL, M (SD) | 43.64 (16.1) | 39.34 (14.31) | t = 1.76 | 151.4 | 0.081 |
| Baseline PH AAQoL, M (SD) | 48.21 (13.74) | 46.01 (12.41) | t = 1.89 | 151.4 | 0.061 |
| Baseline LO AAQoL, M (SD) | 43.04 (19.21) | 37.5 (17.15) | t = 1.05 | 151.6 | 0.297 |
| Baseline R AAQoL, M (SD) | 53.92 (19.64) | 50.05 (20.44) | t = 1.20 | 150.7 | 0.233 |
| Age, M (SD) | 33.4 (11.5) | 32.7 (10.8) | t = 0.75 | 290.4 | .453 |
| **Categorical variables (chi-squared test)** | | | | | |
| Female sex, n (%) | 214 (46%) | 84 (53%) | χ²= 1.70 | 1 | .193 |
| Treatment group PsyEd+CBT, n (%) | 219 (48%) | 78 (49%) | χ²= 0.06 | 1 | .806 |
|  | | | | | |
| **Block 2. Logistic Regression: Predictors of Response Status** | | | | | |
| **Predictor** | **OR** | **95% CI** | **SE** | **z** | **p** |
| Intercept | 0.35 | [0.14, 0.87] | 0.46 | -2.27 | .023 |
| Baseline total AAQoL | 1.01 | [0.99, 1.02] | 0.01 | 0.72 | .472 |
| Baseline LP | 0.92 | [0.62, 1.34] | 0.20 | -0.44 | 0.663 |
| Baseline PH | 0.95 | [0.77, 1.16] | 0.11 | -0.49 | 0.626 |
| Baseline LO | 0.94 | [0.74, 1.18] | 0.12 | -0.51 | 0.610 |
| Baseline R | 0.96 | [0.80, 1.14] | 0.09 | -0.48 | 0.629 |
| Age | 1.00 | [0.98, 1.01] | 0.01 | -0.34 | .735 |
| Sex (male) | 0.75 | [0.52, 1.08] | 0.19 | -1.53 | .125 |
| Treatment group (PsyEd) | 0.97 | [0.67, 1.41] | 0.19 | -0.17 | .865 |
| **Note.** OR = odds ratio; CI = confidence interval; SE = standard error. Response status coded as 1 = respondent, 0 = non-respondent. No statistically significant predictors of response status were identified (all p > .05). | | | | | |

**Table S2**

Post-hoc Pairwise Comparisons of AAQoL Subdomain Scores Across Time Points (LP, PH, LO, R)

|  | **means** | **means** | **t_value** | **DF** | **p-value** |
| --- | --- | --- | --- | --- | --- |
|  | | | | | |
| **LP** |  |  |  |  |  |
| Baseline vs post | 41.54 | 71.57 | -17.24 | 302 | < 0.001 |
| Baseline vs FU | 41.54 | 54.15 | -6.82 | 308 | < 0.001 |
| post vs FU | 71.56 | 54.15 | 9.45 | 305 | < 0.001 |
|  | | | | | |
| **PH** | | | | | |
| Baseline vs post | 40.30 | 65.10 | -12.2 | 301 | < 0.001 |
| Baseline vs FU | 40.30 | 50.00 | -4.6 | 311 | < 0.001 |
| post vs FU | 65.10 | 50.00 | 7.5 | 307 | < 0.001 |
|  | | | | | |
| **LO** | | | | | |
| Baseline vs post | 47.14 | 66.86 | -12.95 | 301 | < 0.001 |
| Baseline vs FU | 47.14 | 41.64 | 3.28 | 300 | 0.001 |
| post vs FU | 66.86 | 41.64 | 14.83 | 301 | < 0.001 |
|  | | | | | |
| **R** |  |  |  |  |  |
| Baseline vs post | 52.04 | 72.19 | -9.41 | 297 | < 0.001 |
| Baseline vs FU | 52.04 | 64.50 | -5.62 | 309 | < 0.001 |
| post vs FU | 72.19 | 64.50 | 3.72 | 306 | < 0.001 |
|  | | | | | |

**Table S3**

*Multiple Ordinal Regression: Predictors of Clinically Relevant Quality of Life Improvement*

**Table S4.** Sensitivity Analysis: Linear Mixed-Effects Model Fixed Effects Estimates Following Multiple Imputation (m = 20)

| **Fixed Effects Estimates After Multiple Imputation** | | | | | |
| --- | --- | --- | --- | --- | --- |
| **Predictor** | **Estimate** | **SE** | **95% CI Lower** | **95% CI Upper** | **Missing Info** |
| (Intercept) | 46.62 | 1.31 | 44.05 | 49.18 | 2% |
| Treatment group (PsyEd+CBT) | -3.64 | 1.88 | -7.32 | 0.04 | 3% |
| Time (Post-Treatment) | 22.64 | 1.73 | 19.25 | 26.03 | 8% |
| Time (Follow-Up) | 5.95 | 1.67 | 2.68 | 9.22 | 1% |
| Treatment × Time (Post-Treatment) | 1.85 | 2.45 | -2.96 | 6.66 | 7% |
| Treatment × Time (Follow-Up) | 2.57 | 2.39 | -2.11 | 7.25 | 2% |
| **Note.** Results are based on multiple imputation with m = 20 imputed datasets using predictive mean matching. Estimates were combined using Rubin's rules. SE = standard error; CI = confidence interval. Missing Info reflects the proportion of variance attributable to missing data. The reference category for treatment group is PsyEd; the reference category for time is Baseline. No significant treatment or interaction effects were observed. | | | | | |

**Figure S1** Diagnostic Plots for Linear Mixed-Effects Model Assumptions: Residuals and Random Effects
